# The New Field Ambulance of the British Army

**Published:** 1907-09-21

**Authors:** 


### The Royal Army Medical Corps Section.

### THE NEW FIELD AMBULANCE OF THE BRITISH ARMY.

The leading features of the new field ambulance of the British Army, as laid down in the correction slip for the R.A.M.C. Manual issued with Army Orders on March 1, 1906, merit attention. Each complete field ambulance of the new type accommodates 150 sick or wounded in tents, and has attached to it a transport equipment of ten ambulance wagons, nine general service wagons, and three water-carts. Its personnel numbers 192 officers and men of the R.A.M.C., and 60 non-commissioned officers and men of the Army Service Corps for transport duties.

The first principle kept in view in the organisation of the field ambulance has been to combine in it the two elements of the Bearer Company and the Field Hospital of the old organisation. This has been done by having in the field ambulance two main divisions, namely, the Bearer Division for stretcher work and the Tent Division for hospital duties, the former numbering 124 men and the latter 68. These are not, as previously, separate commands, both units being now under the medical officer in charge of the field ambulance, and entirely under his control in every detail. Another important point provided for in the new field ambulance is that it has been adapted to meet the varying conditions of warfare. In every campaign operations have often to be carried on by small bodies of troops whose numbers would not require a complete field ambulance to accompany them. To meet this not unlikely contingency the field ambulance has been made divisible into three sections, each complete in itself in both personnel and equipment, and capable of being moved, or even mobilised, independently of the others. In each of these sections is represented the Bearer (stretcher) and Tent (hospital) Divisions, so that each section is practically a miniature field ambulance, and works as such. The three sections are named respectively "A," "B" and "C," but "A" is known in addition as the Headquarter Section, as the commanding officer attaches himself to it. It has some few extra administrative duties to perform, but in all other respects it is identical with its fellow sections.

It was mentioned above that the personnel of a field ambulance numbers 192 officers and men of the R.A.M.C., but it should be pointed out that this is only when the field ambulance has taken the field, and is at the front. On mobilisation—that is to say, when it is raised to its war establishment of men, horses, and equipment, so as to be in full working order for the duties it has to perform—it has really 209 officers and men of the R.A.M.C. attached to it, but of these there are left at the base a storeman and a reinforcement of 16 men to replace casualties. Referenc, too, should be made to another detail in mobilisation that is of some importance, as it materially affects the equipment provided. The mobilisation of a field ambulance may be ordered under one of two scales. Under the first it is assumed that tents are required only for the sick and wounded, the personnel of the field ambulance bivouacking in the open just as the rest of the combatants. On the other hand, under the second scale the personnel are to be provided with shelter, this latter taking the shape of extra tents or of S.S. (special service) blankets, furnished with eyelet-holes, and capable of being erected into shelters by means of small wooden poles. For this additional equipment extra transport vehicles are, of course, provided. It follows, as a matter of course, that the introduction of this new field ambulance has altered the arrangements for rendering assistance to the wounded in war. To begin with, the old brigade organisation, under which a field hospital was attached to each brigade, has been abolished, and the new scheme provides two field ambulances for each division of infantry, the cavalry being provided for by a specialised form of field ambulance. As each field ambulance can accommodate 150 patients provision is made with each division for 300 sick and wounded, the same number as were provided for under the old regime of three field hospitals to a division, each field hospital being considered able to deal with 100 patients. The new plan seems a preferable one, as under it there is more elasticity and convenience than with the old field hospital, for the two divisional field ambulances with their six sections really furnish six distinct, but yet completely organised medical units, any one of which is capable of dealing with 50 patients, and is ready for immediate service. This is a matter of no small moment, as it permits of the wants of a mobile force being at once provided for.

Coming to the actual duties in the field of the new field ambulance, we find that several very important modifications have been introduced. One is that the wounded are to be moved about as little as possible. Under the old arrangement of Collecting station, Dressing station, and Field hospital in the rear frequent changes from stretcher to wagon and from wagon to stretcher took place. These transferences have been minimised to a great extent, the new principle established being to bring the tents to the wounded rather than the wounded to the tents. Hence, there is no longer a recognised collecting station, though circumstances will, of course, more or less establish one, and the ambulance wagons follow close on the stretcher squads of the Bearer Division of the field ambulance. The only fixed station is the Dressing station, which is put as far forward as possible consistently with safety to the wounded, care being taken that it is sheltered, that it is in close proximity to a road and to a good supply of reliable water, and that there is sufficient space near to allow of the whole field ambulance being encamped when the dressing station is moved forward.

This principle that the wounded should be moved about as little as possible has very properly, we think, been specially extended to severely wounded men, such as cases of compound fractures of the thigh and penetrating wounds of the abdomen and chest. For these hand-carrying is preferable to the jolting of an ambulance, and it is laid down that patients suffering from such injuries are to be carried on stretchers to the dressing station. A change such as this throws more work on the stretcher bearers, and for this reason probably an increased number of men is assigned to each stretcher, six doing duty with it in the new scheme instead of four as formerly. In this way a more frequent change of bearers is provided for. As yet no formal drill book embodying these changes has been issued, but the training at Aldershot is at present being carried out on these lines, and the method adopted of dealing with the two extra men is to place one in front of the stretcher squad and in charge of it, while the other one is posted behind. The object of this arrangement is that the leading man is in a position to choose the easiest and best route for the stretcher bearers to follow. From what has been said it is clear that the introduction of the new field ambulance has led to changes in the scheme of assistance to the wounded in war that are of a beneficial kind, and are likely to lessen suffering and save life. The only weak point in the new scheme is the provision of the necessary transport personnel. This should be furnished by the R.A.M.C. itself, as is done in the R.A.M.C. (Vol.), and not by the Army Service Corps. In this way only will this important detail be satisfactorily supplied, and the R.A.M.C. made, as it should be, an independent working branch of the Army.

